# Returning Serve in Tennis: A Qualitative Examination of the Interaction of Anticipatory Information Sources Used by Professional Tennis Players

**DOI:** 10.3389/fpsyg.2018.00895

**Published:** 2018-06-07

**Authors:** Georgina Vernon, Damian Farrow, Machar Reid

**Affiliations:** ^1^Institute of Health and Sport, Victoria University, Melbourne, VIC, Australia; ^2^Game Insight Group, Tennis Australia, Melbourne, VIC, Australia

**Keywords:** tennis, sport expertise, anticipation, decision making, performance psychology

## Abstract

Research has largely focused on the individual contribution of either kinematic or contextual information sources to the anticipatory skill of an expert athlete during a time-stressed situation. Very little research has considered how these two sources of information interact with each other to influence anticipation. The current study used a qualitative interview methodology to investigate this interaction. Eight former or current top 250 professional male tennis players participated in a 30–60 min interview about the interaction of kinematic and contextual information sources and their influence on anticipation. Using an open-coding analysis approach, codes were identified by each researcher from the transcribed interviews and then brought together to identify common themes. The primary themes were consciousness, tactical awareness, contextual information sources, kinematic information sources, mentality/confidence, returner technique or strategy, and build pressure on the server. Secondary themes coded from the participants were returning characteristics and practice. Consequently, a temporal model was developed which demonstrated the sequence and interaction of both kinematic and contextual information sources known to influence expert tennis player’s anticipation.

## Introduction

In professional sport, one skill that sets the experts apart from the novice athletes is the capacity to more efficiently anticipate, react, and move in response to game situations (Cañal-Bruland and Mann, 2015). Anticipatory information is available in the form of kinematic and contextual information sources that become available to a performer at various times prior to an opponent making contact with the ball in time-stressed game situations. How such information influences anticipation skill in expert and novice athletes has been of interest to sports scientists for some time (Müller and Abernethy, 2012). While the results of such work have demonstrated that experts display superior anticipation compared to novices (Mann et al., 2007), current research has largely failed to consider how both kinematic and contextual information sources are integrated or prioritized by an athlete (see Schläppi-Lienhard and Hossner, 2015 for an exception).

In interceptive sports, such as tennis, the most widely examined source of anticipatory information has been the kinematics presented by an opponent (Goulet et al., 1989; Jackson and Mogan, 2007). For example, a tennis server may serve with a ball toss which reaches a zenith more on the left side of a right-handed player than the right which suggests that a wide serve is the most probable serve due to the kinematic constraints of that action (Reid et al., 2011). The influence of kinematics on anticipation has been supported by anecdotes of professional players, whom have variously attributed their success on return of serve to being able to extract meaningful information from the service actions of their opponents. For example, one of the game’s current best returners, Andy Murray, attributed an upset loss to the speed of his opponent’s arm action on serve which made it difficult to pick up (Schlink, 2017). The efficacy of specific kinematic information sources predictive of serve direction have been empirically examined using a combination of temporal and spatial occlusion methods (Farrow and Abernethy, 2003) and gaze-tracking (Goulet et al., 1989). The ball toss, trunk rotation, and arm rotation are all suggested to be important information sources used by an expert returner that lesser skilled performers are not attuned to (Singer et al., 1996; Ward et al., 2002; Jackson and Mogan, 2007).

Anticipatory responses informed by contextual information sources have also been examined (Crognier and Féry, 2005; McRobert et al., 2011; Farrow and Reid, 2012; Loffing and Hagemann, 2014). Contextual information sources relate to the “probabilistic information that is independent of the observed movement and the visual information from the observed movement” (Cañal-Bruland and Mann, 2015, p. 1). That is, contextual information describes all non-kinematic information sources present to help athletes anticipate an opponent’s action. This includes information such as the game situation i.e., the score or an opponent’s court position, an opponent’s perceived strengths and weaknesses in addition to external factors such as wind direction or court surface. The influence these contextual information sources have on anticipation has been examined in a variety of ways. For example, (Farrow and Reid, 2012) manipulated the probability of tennis service direction based on the score and found older more skilled players were more attuned to this information and were able to prepare their response earlier than younger less skilled players (see also Loffing et al., 2015). Similar findings have been demonstrated in other sports such as baseball, where contextual information sources based on particular pitch count scenarios influences the type of pitch to be thrown (Cañal-Bruland et al., 2015) and how batters handle this “count” information (Paull and Glencross, 1997). Alain et al. (1983) were one of the first research teams to examine the influence of contextual information or the phenomenon of players building knowledge of their opponent’s previous shot into their decision making. Alain et al. (1983) reported that as participants perceived the probability of a shot occurring to increase, so too did their number of biased preparations. This manifested itself in the players “setting-up” for the shot they most expected to receive in advance of their opponent striking the ball. Alain et al. (1983) demonstrated that athletes who were aware of the situational probabilities of events occurring responded faster to the more likely event. In the context of the current study, these results highlight the salience of contextual information in the decision-making processes of skilled players in addition to kinematic information available to them.

While a great deal has been learned from the collective body of experimental work that has considered the influence of kinematic and contextual information sources, there has been relatively little investigation into how a performer may selectively use both information sources, despite this being the norm in the performance setting (Cañal-Bruland and Mann, 2015). Schläppi-Lienhard and Hossner (2015) utilized a qualitative approach to address this issue as it related to the decision making of expert beach volleyball players during defensive actions. They considered the respective contribution of visual perception skills such as gaze behavior as well as domain-specific knowledge such as tactical insight and opponent strengths and weaknesses. A key finding from this work, was the prioritization players gave to different information sources dependent upon the situation. For instance, when the situation was largely predictable or as expected by the player they tended to rely on their tactical knowledge, whereas in situations where an opponent was out of position and needing to adapt, they tended to analyze the specific situation by reading their opponent’s movements. The richness of information captured by Schläppi-Lienhard and Hossner (2015) demonstrates the value in using a qualitative research approach to investigate the complex interaction between contextual and kinematic information sources. Consistent with the extant quantitatively focused literature on anticipation (e.g., Farrow and Reid, 2012), domain and task specificity is likely to be a prominent influence on any qualitative insights collected. Consequently, the current study sought to extend and generalize the findings of Schläppi-Lienhard and Hossner (2015) within the sport of tennis through examination of the most influential situation in the game, the return of serve. Through the specific exploration of the interaction between kinematic and contextual information sources on anticipation in tennis decision making, it is suggested a framework for future quantitative research to selectively manipulate and examine the influence of both information sources *in situ* can be provided. This in turn can then provide empirical support to the insights offered by expert performers.

In summary, the extant research has largely focused on the kinematic or contextual influences on anticipatory performance in isolation from other (Cañal-Bruland and Mann, 2015). It is argued that we need to consolidate our understanding of these two information sources to better inform the future study of anticipation in time stressed sport situations. Hence the current study sought to determine the information sources expert or former expert professional tennis players used and prioritized to help them anticipate serve location, type and speed when returning in tennis matches. To achieve this aim, a semi-structured interview approach was adopted, as such techniques are increasingly being used in athlete and coach settings to provide a deeper understanding and perspective on how such information sources are used in time-stressed sport situations (Weissensteiner et al., 2009; Schläppi-Lienhard and Hossner, 2015). It has been argued that this method of naturalistic inquiry can generate deeper insights into the explored question over quantitative research which may exclude certain information needed to explore this research empirically (Pitney and Parker, 2001) such as consideration of the wider influences on an athlete’s anticipation performance due to psychological and physical factors.

## Materials and Methods

### Participants

The participants were eight (8) former (*n* = 6) and current (*n* = 2) Association of Tennis Professionals (ATP) male international tour players known to the research team through their work for the national tennis association. Four of these athletes were regarded as being renowned for their return of serve skill, while four athletes were known more for their serve skill (as identified in media by expert tennis coaches and commentators). Peak career singles rankings of the players ranged from 44 to 152 (*M_rank_* = 75.38, *SD* = 46.02) in the world. Participants at the time of interview were aged from 27 to 55 years (*M_age_* = 41.75 years, *SD* = 9.53) and had competed professionally for 7–18 years (*M_comp_* = 13.50, *SD* = 3.85), between 1978 and 2017, with a mean number of professional singles titles of 4.88 (*SD* = 5.57). Former and current players were not treated separately in the analyses as the interview context was focused on their reflections as a player.

### Procedure

Participants were invited to complete a semi-structured interview that ranged from 30 to 60-min where they detailed the key factors they considered when anticipating a serve during their professional careers. A suitable time and place was organized to meet with the researcher/s where the interview could be conducted without distraction. The interviews were recorded using an Olympus VN-741PC digital voice recorder. Interviews were then transcribed verbatim to be used for analysis. Prior to the interviews commencing, each participant had reviewed an information sheet concerning the purpose of the research and signed a consent form. Institutional ethical approval was granted prior to the study commencing.

### Interviews

The interview guide was developed by the researchers prior to the interview period commencing. The main bulk of the interview guide asked each participant the same broad questions and was relatively unstructured. This was to allow for probes and follow-up questions to the participants responses in order to gain an in-depth understanding of what the participants were discussing (Hardy et al., 2017). This method of interviewing allowed the responses from the participants to be consistent across all interviews, but also allowed the participants to discuss and interpret the questions in their own way (Mishler, 1986). This method of interviewing meant that the researchers had to probe and clarify pieces of information given to them by the participants to ensure all aspects of the questions had been covered, however, it also allowed the participants freedom to answer the questions in their own style. After piloting the interview questions with two coaches who were also retired professional players (though not at the level of those included in the final sample) the finalized interview guide can be found in **Table 1**. In addition to the questions asked, it should be highlighted that the second component of question three was particularly critical in addressing our primary research aim and was probed more extensively than other questions to ensure any content the participant possessed about how the two information sources interacted was collected. Probes were used at the end of each question as necessary to gather further details about the answer.

**Table 1:** Interview guide for participant interview questions.

Q1	Can you think of examples of past or present players who are/were good returners?
Q2	When you were returning, did you consider contextual information (e.g., score, court side, handedness, wind etc.) to help predict an opponent serve when competing?
Q3	What role did kinematic information (e.g., ball toss, trunk rotation, head position etc.) play in helping you anticipate or predict the serve? Was it more important than the contextual information? Why?
Q4	If you utilized such contextual information how did you update it over the course of a match? For example, how many times did a player have to serve to the same spot on big points before you considered this a trend and adjusted your response accordingly?
Q5	Today, we have asked you to think back to when you played. Now that you’re more involved in coaching, has your thinking or philosophy regarding what factors are important on the return changed at all?
Q6	Given the role of the return in tennis, can you comment on how it is practiced?
Q7	Anything else you would like to add about the return of serve and what we have covered today?

### Analysis

Using an open-coding analysis approach (Straus and Corbin, 1998), the transcribed interviews were individually coded sentence by sentence by the three researchers separately to draw upon the emerging themes from the participants responses to the interview questions. The three researchers who conducted the analysis have both research and practical experience in skill acquisition and tennis analysis which was useful in being able to extract and interpret tennis specific jargon from the transcribed interviews. Sentences of the interviews were given tags which related to codes which emerged during the coding process. Themes were included based on common tags from each researcher which were mentioned multiple times in the interviews. Common themes that emerged from this process between each interview were categorized into higher or lower order themes. Some tags resulted in an accumulation of similar meaning labels and were categorized into the same relevant theme. The resultant codes from the thematic analysis from each researcher were drawn together to determine like themes across the researchers (for the count of each theme that is explored in the interview process, see results section). In the case of differing themes between the researchers, findings were compared and discussed, and where appropriate, re-analysis of the related tags was undertaken until consensus was achieved among all researchers. Quotes from the interviews were extracted to provide examples of responses which related to each higher and lower order theme and provide evidence that the themes were relative to returning serve in professional tennis matches. Using this approach allowed the researchers to build upon the anticipation research already conducted by elaborating on the quantitative results of previous studies in anticipation. A grounded theory approach was used to then develop a model which combined the emerging themes of the current study with known data from previous research (Straus and Corbin, 1998). Specifically, Farrow and Abernethy (2003) used viewing windows of 300ms intervals in their temporal occlusion research of a tennis serve. Each 300ms time intervals is known to contain important anticipatory information and will be used as the template for the temporal model developed in this study.

## Results

The thematic analysis which occurred from the transcribed interviews generated nine higher order themes relating to use of specific anticipatory information sources during the return of serve process in tennis matches. These themes were generated using an aggregation of terms and codes from the analysis of the interviews. Importantly, the labels used were based upon the participant’s language rather than a strictly scientific language. Throughout the results, counts of the number of participants who discussed each theme is also included. The themes which emerged describe both a temporal approach and specific informational content used by players to inform their decision making as they attempt to return the serve of their opponent. Each of the themes is summarized in **Table 2** and then further detailed below. The results are presented in two sub-sections. The first section reports the themes that were derived from the participant’s first-person perspective as a returner, while the second section reports themes that were considered subjective theories about the behavior of returners more broadly. Similarly, while the primary aim of the work was to understand return of serve anticipation, participants were also invited to discuss the return of serve more broadly. This discussion and resulting themes contributed to contextualizing the overall research question.

**Table 2:** Nine higher order themes and the corresponding lower order themes resulting from return of serve anticipation interviews.

Higher order theme	Lower order themes
Consciousness	**Anticipation:** *See the ball early off the racquet* *Look for signs/information sources about the type of serve* **Pattern recognition:** *3 service games/halfway through first set to recognize patterns* **Watching pre-match, warm-up and during match opponent’s strengths/weaknesses, figure out information sources and update information** **Some “gut” returning in matches (early)** **Interaction of conscious and non-conscious returning** **Awareness of contextual and kinematic information sources** **Limitations of server:** *Handedness* *Weaknesses*
Tactical awareness	**Awareness of contextual and kinematic information sources** **Calculation about what serve is coming** **Playing the percentages** **Constantly updating probabilities** **Handedness:** *E.g., Left-handed servers preference sliders on Ad court*
Contextual information sources	**Server preferences:** *On score lines (e.g., break point, 30–30)* *Court side* **Weather:** *Wind conditions across/down court* **Surfaces:** *Grass* *Indoor* *Clay* **Left v right handed servers** **Situational information** **Court slope** **Play percentages but must be aware of the situation in the moment**
Kinematic information sources	**Server position on baseline** **Ball toss** **Server’s grip** **Torso rotation** **Server action:** *Shoulder over shoulder* *Corkscrew* **Body position when driving up – serve-volley**
Mentality/confidence	**Making returns early in the match** **Clarity of returning:** *Confidence in execution of returns* **Critical in being good returner** **“The bluff”** **Switched on/focused**
Returner technique/strategy	**Return to large targets/locations on court** **Adapted swings:** *Compact swing* *Grip changes* *Double handed backhand better than single handed backhand for returning* **Protect returner’s weakness** **Confidence in returning ability (backs self)** **Ball tracking/recognition** **Hits return across body**
Build pressure on server	**Making a lot of returns to force server to over-serve** **Feel presence of returner** **“The bluff”:** *Returner moves laterally/forward/back before serve to get inside server’s head* *Shows the server that as a returner, you know where the serve is going*
Returning characteristics	**Aggressive returners:** *Stands up in court* *Returns well on big points* *Tees off* **Neutral returners:** *Returns serve-by-serve* *Just aims to get ball back into court* **Counter-puncher returners:** *Makes high percentage of returns to increase pressure on server* *Not aced a lot* **Consistency** **Fast feet** **Picks up information sources/signs/ball earlier** **Quick hands to recognize the serve and adjust grip/racquet position to hit quality return** **Agility:** *Explosive* *Quick feet* **Take ball early** **Forward momentum** **Set-up:** *Adjust returner’s court position relative to server’s*
Practice	**Not practiced enough** **Not specific enough** **Best practice via exposure to different servers, serves, handedness, ball tosses** **Doubles useful for gaining tactical experience**

## Anticipation Perspectives

### Consciousness

All eight participants agreed that they were conscious of the various information sources they needed to look for in order to anticipate and correctly decide the type of serve they needed to return during a match. The notion of conscious detection of these information sources was a common theme throughout all interviews. When discussing the detection of kinematic and contextual information sources of a server, participant 6 said “if you know a guy prefers a certain serve on a certain point… then you can take a calculated risk or a guess that you can maybe sit a little more on that one. But personally, I also get a feel and a read for guy’s techniques and I’m able to see pretty quickly which serves they’ll be able to hit at a higher percentage when they really need them according to their technique.” This comment shows that tennis players are consciously aware of various contextual and kinematic information sources which would result in a particular outcome that would help them anticipate particular types of serves. This conscious gathering of information would continue during the match and be constantly updated based on new information and information sources from their opponent throughout the match. All eight participants were in agreement that “if you’re switched on enough you can probably work out in the first two or three service games” (participant 8) the kinematic and contextual information sources of their opponent if they had not each other before. All eight participants suggested that this collection of information may also occur in the days or hours prior to the match. This includes information about the server given to them by a coach, other players, by watching their opponent’s previous matches, or during the warm-up.

### Tactical Awareness

Being tactically aware about the high percentage plays used by a server also demonstrated that the players were conscious of potential contextual information sources and kinematic information sources that they could draw upon to help anticipate the direction and location of a serve. Respondents in the interviews frequently spoke about updating probabilities of certain serves throughout the course of a match and making calculations about what serve they were about to return. “Statistically, if you know a guy prefers a certain serve on a certain point, or a big point, say key points in the matches, then you can take a calculated risk or a guess” (participant 6). Having good tactical awareness when returning a serve meant being aware of the many contextual and kinematic factors which would contribute to anticipating a high percentage situation. Participant 4 said that “you’ve got to know, particularly on big points, break points, crucial points, the more important points in the game, you’ve got to be very very aware of what your high percentage plays are.”

### Contextual Information Sources

All eight respondents cited that they used various contextual information sources to help anticipate a serve in tennis matches, and that it only took two or three service games from their opponent for them to be aware of the server’s preferences. The most common contextual information source participants used was known server preferences on score (most predominantly, on break point or game point). Participant 1 discussed that “on a big point, they [the server’s] are going to want to hit their favorite serve most likely. I’m going to make sure I don’t get aced by that, I’m going to try and cover that favorite one at least.” Additionally, other contextual factors which they considered when returning serve were factors which could not be changed or updated prior to or over the course of a match, such as weather conditions, court surface, indoor, or outdoor conditions, handedness of server and the court slope. Each of these factors was listed by five of the eight participants as factors which needed to be carefully considered when playing a match. Participant 8 spoke of the how different court surfaces affect the serve and the return “on clay, you probably see that a little bit more where they maybe go out to the backhand side, they hit a kick serve generally. They want to get more angle to hit a forehand off the next ball.” One criticism of using contextual information sources to return serve by some of the respondents, was that, although they were always aware of the probabilistic information at a given time during the match, this information would not negate the other information they were aware of at the time, such as kinematic information sources. Participant 5 described this as “I think that obviously, myself, when you’re up against a big point, you want to go to your favorite serve…You can’t take that as religion, but it does help to know that they’re more likely to go there.” In most cases, it appeared that the kinematic information was the factor which either confirmed or changed what the returner knew from the contextual information.

### Kinematic Information Sources

Kinematic information sources were commonly mentioned by all participants when anticipating a serve during a match. The most common factor mentioned by the eight participants was the ball toss of the serve; participant 4 said that “I suppose I would look mainly at the toss. The action generally from player to player isn’t going to change that much. The toss obviously changes, so you’re trying to get little cues out of the toss.” Other kinematic factors that were mentioned were the server’s grip on their racquet, the server’s position on the baseline, torso rotation during the serve, and the type of service action of the server. Two types of service action were mentioned by participant 5 who described it as “the old school corkscrew service action, which is where they get their power and their rotation, isn’t shoulder over shoulder, it’s torque rotation where your shoulders are actually moving in a semi-circle almost. When that happens, I feel like that those guys are a lot more susceptible to cut, have good cutting serves, sliding the ball. Shoulder over shoulder, they have much more ability to hit that flat serve.” These discussion points makes evident that kinematic information sources are very much focussed on during the return of serve.

### Mentality/Confidence

In order for tennis players to anticipate a serve and use the anticipatory information, they must be confident that the information they are perceiving is sufficiently reliable for them to act upon before ball flight information is available. Six of the eight respondents said that being focused on the anticipatory information sources they were looking for, was key for having the confidence to execute a return from an anticipated serve. Participant 8 said it was important for players to have clarity in their decision making when attempting to return a serve. Participant 8 described that “it really comes back to preferences under pressure for a lot of these players. And then having the ability to back that as well. You can say that you’re going to do it, and know that it’s going to happen, but then you’ve actually got to try and be leaning that way, and actually have the conviction in your head that that’s the way he’s going to actually serve it”. Another strategy participant 7 discussed to build confidence in returning ability was to execute these types of serves early in the match, so the returner had already experienced the types of serve they may be expecting when it was most crucial (i.e., on break points or late in a set). For example, “early in a match… I would always hit a return up the line early to free up because when it gets tight, it’s harder to hit the more difficult returns. If you haven’t done it, then mentally you won’t take it on, you’ll go back to safety.”

### Returner Technique or Strategy

An effective returning technique that emerged from the responses was to ensure that the returner had compact swings off both the forehand and backhand sides (due to the time constraints of a returning task) as well as aiming to large targets or locations at the other end of the court. Participant 3 compared hitting a return to hitting a baseball: “not taking big swings. I refer to it a little bit as, sometimes on a big serve you can bunt the ball like a baseball where you don’t take a big follow through because when the ball’s coming really fast and you’re taking a complete swing, there’s more chance of an error.” Additional strategies include hitting the return across the body so it passes over the lowest part of the net, protecting the returner’s own weakness, and being confident in their ability to hit the return how and where they wanted to. As participant 6 said, “I think statistically, there’d still be some foundational things that work better than others, or history would show things work better than others, for example, returning over the lower part of the net, or returning to big spots in the court.”

### Build Pressure on Server

Many returners say that they would prefer to hit a second serve as opposed to a first serve in a match as second serves are often more predictable and much slower than a first serve. While this is very server-determined, participants discussed the tactic of forcing pressure onto the server to attempt to bluff them into serving either a slower-paced first serve or a second serve. Participant 3 said that “second serves are notorious where people can get a bit nervous on them and serve double faults so if you can play with their head a bit, you can get a few free points, so that was always important, or get them to start slowing down their first serve because they’re worried on big points, of hitting a second serve.” Using kinematic and contextual information sources to build pressure on the server allows returners to position themselves in a way which shows the server that they know which direction the serve will be coming. This tactic forces perceived pressure onto the server who now either has to block out this information from the returner and serve as they were planning to or adjust their serving tactics and change serve direction. Participant 5 said that “I want the server to think about me returning as much as possible. Because anything that will slightly throw them off that rhythm slightly on that serve will be the difference of getting a second serve on a big point as opposed to a first serve. I’m a big fan of getting in the head of the server if I feel like they’re winning that battle. So, I’ll definitely stand around, and that’s where that [statistical] data comes into play. I might go and really show him that I think that down the tee serve is your favorite serve.”

## General Participant Observations

### Returning Characteristics

Three types of returners were described by the participants. Aggressive returners were able to set the point up with their return, a high starting return position in the court (i.e., inside the baseline) and were able to build pressure on the server by doing so. Participant 2 described aggressive returners as “Its not so much it comes at your toes every time, it’s just the fact that you never get any free points. The accumulation of pressure on the server developed by very good returners on their second serves is pretty telling.” Counter-puncher returners have the ability to build pressure on the server, similar to the aggressive returner, however, they did this by making a high percentage of returns back into the court and were said to be more consistent. This type of returner was described as being very frustrating to play against as the server was unable to win a free point from an ace or unreturnable serve. These two returner types were summarized nicely by participant 7 who said “the returners who hit aggressive and who (have) the ability at making good returns at set points or on big points. And then there’s the returner who makes every ball back into play and is more of a counter-puncher returner.” The third type of returner was a neutral returner who was characterized by their ability to simply return each serve back into the court on its own merits without considering kinematic or contextual information sources. While three distinct returner types were identified from the responses, there was consensus among all participants who added that adaptability and consistency were important characteristics which good returners must possess regardless of returner type. Participants said that when talking about players reacting to a serve that, “there are some players who will pick up the signs better, have better reactions, they’re sharper, their eyes work quicker picking up balls” (participant 3). This demonstrates that reaction capabilities of players, as well as their capability to anticipate a serve is important to executing a quality return of serve. Good returners in this area were said to have quick, reactive hands which allowed them to react and adjust the grip on their racquet to hit a quality return.

Participants noted that great returners were able to see and anticipate a serve early, but quick movement to the anticipated serve was critical. As participant 7 described, “I think the best returners in the world are the ones that have the ability to take the ball on the rise and move into the court when it’s a bigger point.” This also requires returners to have good forward momentum moving into the court when returning. “Best returners play the ball. And that fundamentally is a very simple breakdown of body weight forward into the return, you’ve got a good chance of making it and making a better return because you can get a piece of it with some pace on it” (participant 7). Not only this, but participants also discussed the ability of the returner to use the information sources to anticipate where the serve was going and using their movement and return positioning, they were able to position themselves to hit a quality return.

### Practice

All participants said that the return of serve is practiced proportionately less than the serve and is often incorporated in point or match practice play during training sessions, however, it was becoming a more important aspect of training. “It’s probably one area that tennis players don’t practice enough. They practice their serve a lot, you’ll practice your serve way more than if you go and practice your return of serve. I think it’s something that could be practiced more” (participant 3). Participants did say that in the future, a focus on return of serve was something they would consider in coaching to expose junior players to more variety of serves, serve styles, and handedness to develop their awareness of kinematic and contextual information sources. As participant 4 said “I think we can do a better job of actually practicing different return positions better than what we do. Whilst clarity is a big thing, and you’re going to have your favorite position as such, I think it’s important to be adaptable and be flexible with what you can do as a returner.”

## Discussion

**FIGURE 1:**
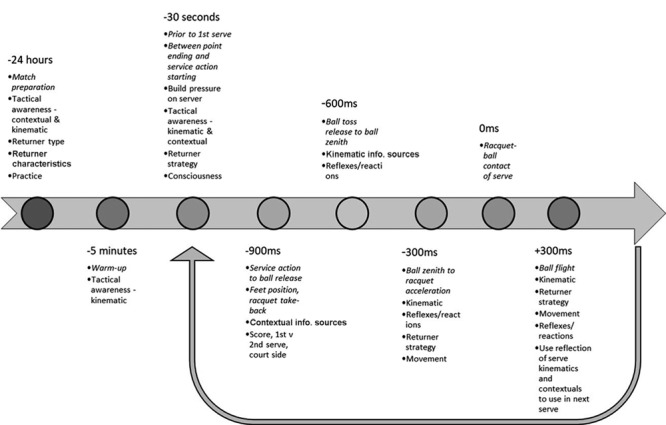
Temporal model depicting the use of anticipatory information sources during the return of serve.

The current study aimed to investigate the interaction of kinematic and contextual information sources on the return of serve. Using an open coding approach to analyze the participant’s responses, nine higher order themes were found from common responses across the participants. These themes were consciousness, tactical awareness, contextual information sources, kinematic information sources, mentality/confidence, build pressure on server, returner technique or strategy, returning characteristics, and practice (**Table 2**). Using a grounded theory approach, these higher order themes and the specific circumstances and types of information considered by the players was aggregated to develop the model which depicts the timeline of serve and return events in a tennis match (**Figure 1**). This model commences with match preparation on the day prior to the match and concludes with the execution of the return shot by the player. A significant feature of the model is its cyclical nature. That is, the actual serve direction and return (type and quality) executed subsequently influence the preparation for the next return of serve and over a number of points and games begin to weight decisions made and which information is used when similar situations are experienced.

The timeline of serve and return events (**Figure 1**) commenced the day before the match, with one respondent suggesting that they would undertake some type of analysis to determine the specific contextual and kinematic information sources unique to their future opponent they needed to be aware of. “You had your coach or yourself watch [your opponent], and say, “look I think they favor this.” But also base it on technique, for example, Boris Becker, he had a forehand grip [on serve], could he go wide to second court? Absolutely, but I still felt like his inconsistency was enough so you might say “well that’s the highest part of the net,” or his forehand grip negated him from have a great swinger down the tee” (participant 1). Returning characteristics was also included in pre-match considerations to allow players to adapt their planned returning strategy based on their returning skill and their opponent. Throughout the match, players have approximately 30 s between the last point ending, and when the server needs to initiate their next service action. Participants said they were able to use this time to prompt themselves of the contextual and kinematic information sources they needed to be aware of during the next serve and undertake an analysis of the updated information sources which have been presented up to that stage of the match. This demonstrates the constantly shifting weighting of contextual information used throughout the match. Having an understanding of the key information sources allowed players to use the between point time to set up their return position with the aim of adding pressure on the server by demonstrating through their positioning, they knew where the serve was going. A good explanation of this was given by participant 7 who said “let’s say on a break point, their best serve was down the tee, [I would] cover more of that and force them to go more to that one that they don’t want to under pressure like that.” This was a conscious decision by the player in an attempt to force their opponent to over-serve, or doubt their serving strategy, resulting in a fault or an easier serve to return.

The commencement of the physical return of a serve goes through distinct stages outlined by the timeline in **Figure 1**. The time intervals presented have been borrowed from conditions used in earlier temporal occlusion research Farrow and Abernethy (2003) where consideration had been given to the kinematic sequence of the service action. The time intervals up to -600 ms are when participants stated that contextual information is used as the predominant anticipation information source. This includes factors such as score, side of the court, weather conditions or server preferences. This is consistent with previous empirical work that demonstrated older, more experienced tennis players are attuned to patterns in serve direction based on score information that younger, less experienced players are not (Farrow and Reid, 2012; Stern et al., 2016). This was nicely illustrated by participant 8 who stated; “You look for patterns in the serving. Obviously, guys under pressure, that’s the biggest key, under pressure where they serve those balls… if you’ve done your homework, generally you can be leaning one way or the other knowing that they’re going to serve that serve under pressure.”

The time period that key information appeared in the event sequence and hence its relative importance in the returners decision making process was summarized by participant 4; “you may know the opponent well. You may have played them before and so that obviously helps to get a bit of a rough guide as to where they’re potentially going to serve on big points. I suppose on top of that, you’re looking at just trying to read little things into their toss. Trying to pick up any cues possible that you can to try to get a slight lean on a serve.” This is a good illustration of how contextual information sources are initially considered in the early stages of the serve (i.e., stages prior to -900 ms), but then the probability of that serve is either confirmed or rejected by the kinematic information from the service action in the time window from -600 ms through to racquet ball contact. This is similar to the model developed by Müller and Abernethy (2012) which demonstrates the early influence of contextual information, however, as an increased number of kinematic information sources become available throughout the service action, anticipation of the action outcome becomes predominantly influenced by kinematic sources.

With the service action phase from -600 ms through to racquet-ball contact, there are a variety of specific kinematic events which are known to contribute to anticipation. These include the lateral position of the ball toss, depth of knee flexion and arm and trunk rotation of the serving player (Ward et al., 2002). The results of this study suggest that expert players consider the ball toss to be the primary kinematic information source used to anticipate a serve. This is demonstrated by participant 3′s comment: “I suppose I would look mainly at the toss…The toss obviously changes, so you’re trying to get little cues out of the toss.” While some previous research Loffing and Hagemann (2014) suggests that contextual information may still be prevalent during these later time intervals, the current results suggest that expert tennis players are seemingly consciously attuned primarily to the kinematic information sources from the opponent’s service action in this period.

Once the server makes contact with ball, clearly the ball flight information overrides all previous contextual and kinematic information. As participant 5 stated, “what I really try to put an emphasis on, is really try to pick it [the ball] up early off the racquet so therefore I can react a little bit quicker.” This aligns with the *in situ* observations of Triolet et al. (2013), who suggested in the majority of cases return responses are largely based on ball flight information. Re-adjusting one’s intent or indeed initial movements based on anticipatory information was also highlighted as a key skill by participants. The returner’s own reactions, movement and returning strategy and execution are considered all critical factors in this regard. Empirical work has demonstrated, that the reactions of returners are faster during the first serve compared to the second serve (Filipcic et al., 2017), which is clearly in part due to the temporal stress of the first serve relative to the second serve. In particular, it has been demonstrated that split step reaction time and movement speed are critical to accurately responding to a fast first serve. Following the completion of a point and before commencement of the next point the returner has time to briefly reflect on the presented kinematic and contextual information sources relative to the actual serve hit and consequently update their predictions for following serves. This process was reported as taking at least three service games for them to feel comfortable that any changes in the likelihood of a particular serve are genuine and not simply due to chance. As participant 8 explained “if you didn’t [know your opponent], sometimes even as much as the second or third service game, because it would be a bit of a test even, because you would take a calculated risk and test them to see if they had the courage to hit certain serves under pressure.” This observation is akin to the early work of Alain and colleagues who suggested that the probability of a specific event needed to be as large as 90% probable before a performer would anticipate its occurrence with confidence (Alain and Proteau, 1977).

While the current study provides some useful insights into how expert tennis players integrate or weight the variety of anticipatory information sources available to them, there were some limitations which must be acknowledged. While the participants in this study met our inclusion criteria, and good saturation of data was achieved, it could be argued that a larger and more diverse sample of experts would provide greater insight. In sports such as tennis, it has often been anecdotally reported that different nations traditionally excel in different styles of play (Davy, 2013) and consequently a broader sample may have overcome this limitation. Further it is also likely that athletes use and are unconsciously influenced by various anticipatory information sources. This may have led to certain factors being under-reported.

## Conclusion

In conclusion, the results of this study provide researchers with a framework to further investigate return of serve anticipation in tennis and quantitatively identify the interaction of kinematic and contextual information sources. The current results generally support previous empirical work that has independently examined the various anticipatory information sources available to a performer. It is envisaged experimental designs can be developed to manipulate the salience of specific information sources and ascertain whether performers do indeed weight or prioritize particular information in specific circumstances. This will allow further insight into the contribution of sub-conscious processing relative to that information performers are consciously attuned to. In addition, the current research findings can be applied using a representative task design approach to develop a training protocol for players to develop their anticipation skills (Broadbent et al., 2017). Similarly, the practical implications of the current study can be extended to interceptive tasks in other sports such as baseball or cricket.

## Ethics Statement

This study was carried out in accordance with the recommendations of National Health and Medical Research Council (NHMRC)′National Statement on Ethical Conduct in Human Research (2007)′, Victoria University Human Research Ethics Committee. The protocol was approved by the Victoria University Human Research Ethics Committee. All subjects gave written informed consent in accordance with the Declaration of Helsinki.

## Author Contributions

GV, DF, and MR all contributed to the conception, design, collection, and analysis of the data, manuscript revision, and approval of final submitted manuscript. GV wrote the first draft of the manuscript.

## Conflict of Interest Statement

The authors declare that the research was conducted in the absence of any commercial or financial relationships that could be construed as a potential conflict of interest.
